# Human gut microbiome aging clocks based on taxonomic and functional signatures through multi-view learning

**DOI:** 10.1080/19490976.2021.2025016

**Published:** 2022-01-18

**Authors:** Yutao Chen, Hongchao Wang, Wenwei Lu, Tong Wu, Weiwei Yuan, Jinlin Zhu, Yuan Kun Lee, Jianxin Zhao, Hao Zhang, Wei Chen

**Affiliations:** a State Key Laboratory of Food Science and Technology, Jiangnan University, Wuxi, P. R China; b School of Food Science and Technology, Jiangnan University, Wuxi, China; c Department of Microbiology & Immunology, Yong Loo Lin School of Medicine, National University of Singapore, Singapore, Singapore; d International Joint Research Laboratory for Pharmabiotics & Antibiotic Resistance, Jiangnan University, Wuxi, China; e National Engineering Research Center for Functional Food, Jiangnan University, Wuxi, China; f Wuxi Translational Medicine Research Center and Jiangsu Translational Medicine Research Institute Wuxi Branch, Wuxi, China

**Keywords:** Gut microbiome, age, metagenomic, machine learning, ensemble, multi-view, regression

## Abstract

The human gut microbiome is a complex ecosystem that is closely related to the aging process. However, there is currently no reliable method to make full use of the metagenomics data of the gut microbiome to determine the age of the host. In this study, we considered the influence of geographical factors on the gut microbiome, and a total of 2604 filtered metagenomics data from the gut microbiome were used to construct an age prediction model. Then, we developed an ensemble model with multiple heterogeneous algorithms and combined species and pathway profiles for multi-view learning. By integrating gut microbiome metagenomics data and adjusting host confounding factors, the model showed high accuracy (R^2^ = 0.599, mean absolute error = 8.33 years). Besides, we further interpreted the model and identify potential biomarkers for the aging process. Among these identified biomarkers, we found that *Finegoldia magna, Bifidobacterium dentium*, and *Clostridium clostridioforme* had increased abundance in the elderly. Moreover, the utilization of amino acids by the gut microbiome undergoes substantial changes with increasing age which have been reported as the risk factors for age-associated malnutrition and inflammation. This model will be helpful for the comprehensive utilization of multiple omics data, and will allow greater understanding of the interaction between microorganisms and age to realize the targeted intervention of aging.

## Introduction

In humans, aging is a continual and progressive process that results in decreased physiologic function across all organ systems. The gut microbiota is considered the most important symbiotic microecosystem, and has multiple functions in human health, including in digestion, immunity, metabolite production, and even neural function.^1^ Numerous cohort studies have implicated age as a dominant factor influencing the adult microbiome.^2^ Given the rapid increase in life expectancy and the increasing proportion of older adults, increased understanding of the biological mechanisms that underlie the gut microbiota–aging interactions implicated in the development of aging-related diseases is warranted. In line with this, the resolution of gut microbiome signatures will be invaluable for the development of noninvasive microbiome-based tests to determine signs of accelerated or delayed aging in the elderly, and to evaluate gut microbiota-based interventions to alleviate the aging-related diseases.

Metagenome-wide association studies have begun to explore gut microbiome alterations in the aging process to investigate the associations of the gut microbiome with age. Microflora evolution does not stop in childhood but extends into adulthood and even old age.^1^ Recent studies have shown that the alpha-diversity of the gut microbiome continues to increase from infancy to adulthood, where it remains relatively stable before increasing again in old age.^3^ Moreover, the proportion of beneficial bacteria, such as short-chain fatty acid-producing species, reduces with age, whereas that of opportunistic species and pathobionts increases.^4^ In addition to the taxonomic component of gut bacteria, functional genes, such as those associated with xenobiotic biodegradation and metabolism, have also been shown to correlate with age, with a more prominent functional rearrangement observed in the elderly.^5^

However, most prior studies have focused on a single cohort, and very few have integrated data from multiple populations or other studies. These limitations hinder our ability to clarify the robustness of microbiome–age associations and obscure our understanding of the potential mechanisms by which the microbiome contributes to aging.

The robustness of microbiome–age associations can be assessed by a meta-analysis of data integrated from all relevant investigations. The investigation of microbiome alterations across a wide age range requires a comprehensive dataset of consistently collated microbiome profiles. Although a deluge of metagenomic data on the associations between the human gut microbiome and age has been generated, obtaining molecular footprints of aging in molecular-level features from these data remains a major challenge. Such findings are disorganized and need to be unified into a theory of gut community dynamics. Machine learning offers next-level analyses that allow the development of new perspectives and novel hypotheses relating to the human gut microbiome. Human microbiome aging clocks are considered a reliable way to measure the passage of time in a gut community and to distinguish two temporally different states. Until now, this pursuit has produced limited results in the gut metagenomics field, despite there being a mass of reports on the association between specific microbes and aging. The ability of the oral, gut, and skin microbiomes to predict age has been previously tested in adults using random forest regression on data combined from multiple publicly available studies.^6^ The results demonstrated that the models based on skin microbiome 16s rRNA data provided the best prediction of age (average mean absolute error [MAE]: 3.8 years; R2, 0.739). However, although these models achieved very good results in age prediction, amplicon sequencing is restricted in its ability to provide an in-depth analysis of the association between microbiome and aging. Besides, in another study, a model based on deep neural network and metagenomics data was proposed (MAE: 5.91 years; R2, 0.29), but the accuracy of its prediction was still lacking and the accuracy was related to the reliability of the final association interpretation.^7^

Although the power of machine-learning algorithms is attracting increasing attention, some limitations remain. First, the complexity of the microbiome and its susceptibility to numerous covariates and confounding variates apart from age complicate the basic task of aggregating the available information into an intestinal aging clock.^8,9^ Second, because metagenomic data are usually sparse and presented as a high-dimensional matrix with high noise,^10^ there is an urgent need for an optimal machine learning method to decipher the links between aging and microflora composition and functions. Furthermore, while previous studies have proven insightful, their focus on taxonomy may limit our understanding of the association between microbiome functions and health.^11^ The ability to resolve the association between microbiome functions and health may prove critical to determining the mechanisms through which the microbiome accelerates or delays aging. Thus, these deficiencies limit the ability to establish a robust age regression prediction model based on gut microbiome metagenomic data, and prevent further understanding of the potential interaction between the gut microbiome and human age. These challenges have led to the introduction of an emerging machine learning field – multi-view learning and envision its potentially powerful applications to gut microflora composition and functions.^12^ In particular, multi-view learning is more effective than previous integrative methods that have been used to learn the heterogeneity of metagenomics data and reveal cross-talk patterns.^12^

The aim of this study was to deliver a universal and reliable aging clock based on the functions and composition of the gut microflora. To this end, we used meta-analysis to rectify the problem of data heterogeneity between multiple metagenomic cohorts based on a large-scale dataset to effectively avoid the influence of host or technical factors. Moreover, we built multi-view learning models to integrate the taxonomic and metabolic pathways of the gut microbiota with the aim to establish the functional and mechanistic interpretations between the gut microbiota and age. Finally, we describe how the abundance and function of microbes affect age prediction, and how this knowledge can be used to identify potential biomarkers of intestinal aging. The results of this study will improve the understanding of the relationship between the gut microbiome and age, provide insights into a new aging-diagnosis strategy, and suggest potential intervention targets for future research.

## Result

### Influence of host-associated factors on the microbiome

**Figure 1.**
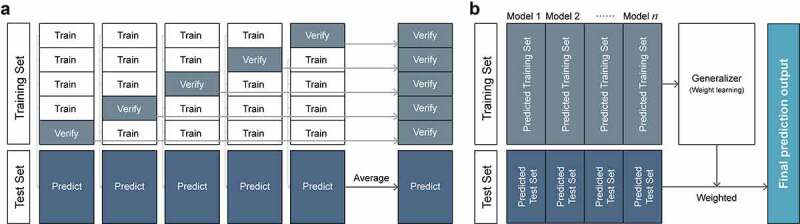
The illustration of the stacking ensemble structure (a) the first stage of model ensemble (b) the second stage of model ensemble.

**Figure 2.**
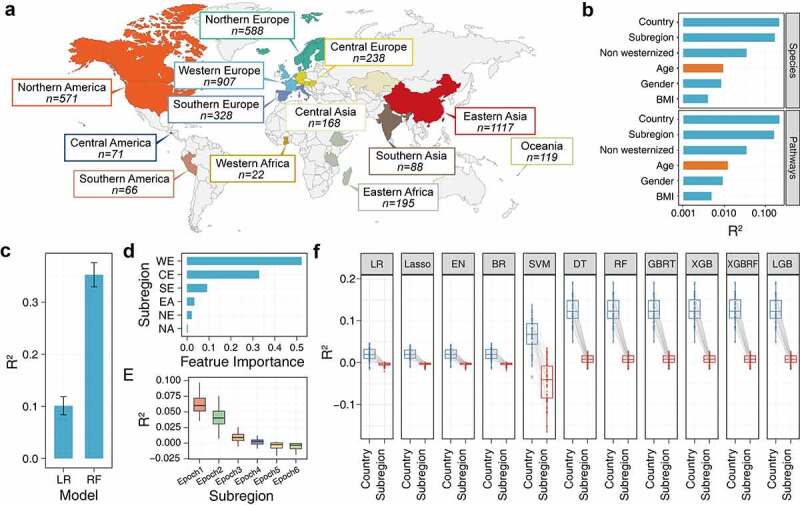
Overview of sample data and the association between host factors and age. (a) The sampling area (regrouped to subregion level) and sample size of the metagenomic data of the gut microbiome used in this study. (b) The effect computed using Adonis after of host factors with microbiome species and pathway composition. (c) The association was computed using linear regression and random forest model of country and age factor. (d) The extent of influence of each subregion on the age distribution sort by feature importance score. (e) The effect between the sampling subregion and age factor of the sample subset during each screening epoch. (f) The effect between the selected sample subsets’ country/subregion and age factors which computed by different algorithms. The performance of all the above models is evaluated by 10 times 5-fold cross-validation.

Abbreviation: WE, Western Europe; CE, Central Europe; SE, Southern Europe; EA, Eastern Asia; NE, Northern Europe; NA, Northern America.

A total of 4478 fecal samples were collected from 31 study cohorts covering 13 subregions (Figure 1a, Supplementary Table S1) that all samples from individuals with age were ≥ 18 years. Permutational multivariate analysis of variance (PERMANOVA) was performed to analyze the influence of age and other host-associated factors on the taxonomic and metabolic pathway profiles of the gut microbiome. Geographical factors, including country and subregion, had the largest interaction with gut microbiome composition and function, followed by Westernization and age (Figure 2b). Collectively, these results showed that age was a major factor responsible for the significant difference in the adult gut microbiome (adjusted for DNA extraction kit and sequencing platform; Bonferroni-corrected *p* < .001).

We next investigated whether the distribution of geographical factors was age-oriented. In general, the nature of the age distribution trend between subregions leads to a decrease in the performance of the age prediction model, and it is difficult to demonstrate that the age-related markers are not driven by geographical factors. Moreover, given the differences in distribution characteristics between countries and ages, addition of the geographical factor as an extra feature to the prediction model leads to a false positive situation owing to the data tendency of the feature itself as opposed to a learned association between the feature and intestinal flora. For these reasons, we utilized two different algorithms (Linear Regression [LR] and Random Forest [RF]) to evaluate the association between country and age. Both algorithms showed that there was indeed a specific trend of sample enrichment in a certain age range in some national datasets (R^2^ > 0.1; Figure 2c), which led to an incorrect correlation between the country and age factors.

We combined two methods to reduce the correlation between geographic location and age to avoid age–regional distribution problems. We first grouped countries into different subregion-level bins based on geographic location; this ensured that, after decrease regions with special distributions, still maintain most samples. After clustering, the subregional factor was still an important factor affecting the species and pathways of the gut microbiome (Figure 2b). And there was still a certain relevance at the subregion level (LR, 0.016 ± 0.007; RF, 0.110 ± 0.019). Therefore, we next screened each dataset on the basis of the clustering result. To ensure statistical power, subregion-level bins with sample sizes > 200 were considered for subsequent analysis. The RF model was used to further judge the strength of the association between subregion and age factors using the feature importance score to sequentially remove the subregion bin that most related to age (Figure 2d). Finally, we identified a subset of subregions that had no obvious correlation with age (R^2^ < 0.01; Figure 2e), including Eastern Asia (EA), Northern America (NA), Northern Europe (NE) and Southern Europe (SE). We evaluated the relevance of this subset on different machine learning algorithms (figure 2f). In all models, the screened data subsets effectively avoided the distribution problem compared to the country level (both R^2^ < 0.01).

### Construction of age regressor models based on taxonomic profiles

The diversity of the base regressor was necessary to achieve an effective model ensemble as heterogeneous algorithms were expected to obtain more diversity, so as to reduce deviation through integration.^13^ Thus, we performed a systematic evaluation of different machine learning algorithms on the adjusted dataset (2604 samples total; all individuals in the dataset were between 18 and 107 years old, with a median age of 52 years).

**Figure 3.**
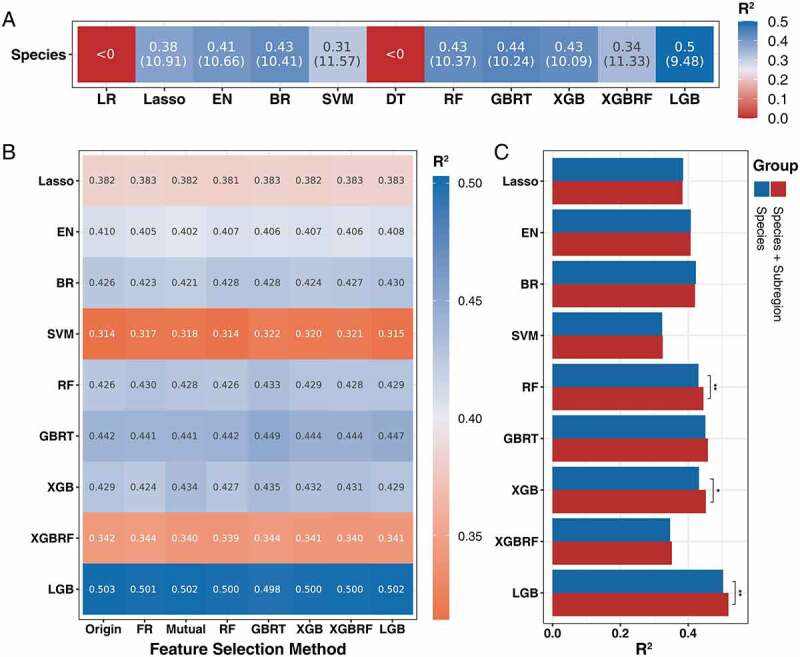
Performance of age prediction model based on species composition of gut microbiome. (a) The ability of different machine algorithms to predict the age of species composition (evaluation metric is R^2^ and MAE). (b) The impact of different feature selection algorithms on the performance of different models (filtered with age prediction ability, evaluation metric is R^2^). (c) The influence of extra subregion feature on the performance of age prediction (based on the species composition after feature selection).

First, we aimed to determine which models were able to achieve age prediction for the gut microbiome species-level profiles. A total of 11 models were considered, including LR, Lasso, Elastic Net (EN), Bayesian Ridge (BR), Support Vector Machine (SVM), Decision Tree (DT), RF, Gradient Boosted Regression Trees (GBRT), eXtreme Gradient Boosting (XGB and XGBRF) and LightGBM (LGB). Except for the LR and DT models, several other models can achieve age prediction (Figure 3a); indeed tree-based algorithms (RF, GBRT, XGB, XGBRF, and LGB) were found to have better predictive performance than other models, and the LGB model had the highest performance (R^2^ = 0.5, MAE = 9.48).

Second, we adopted a series feature selection method with the aim to reduce feature dimensions and decrease computational cost. Among them, the GBRT-based selection method decreased the data feature dimension with no reduction in performance (Figure 3b, Supplementary Table S2). Therefore, we used the GBRT-selected data as the input of the subsequent model ensemble, considering the reduced model calculation overhead.

Additionally, we compared the influence of the extra subregion label feature on model performance based on the filtered data (Figure 3c). The tree-based methods (RF, GBRT, XGB, XGBRF, and LGB) were more sensitive to the additional feature, and the results demonstrated that subregion information can significantly improve model performance.

### Construction of age regressor models based on metabolic pathway profiles

To construct a highly precise age regression model based on gut microbiome metagenomics data and improve data utilization, we further considered the composition of the metabolic pathways of the flora in addition to the previously mentioned taxonomic profiles. Moreover, a systematic evaluation of different machine learning algorithms was conducted on the metabolic pathway annotation results.

**Figure 4.**
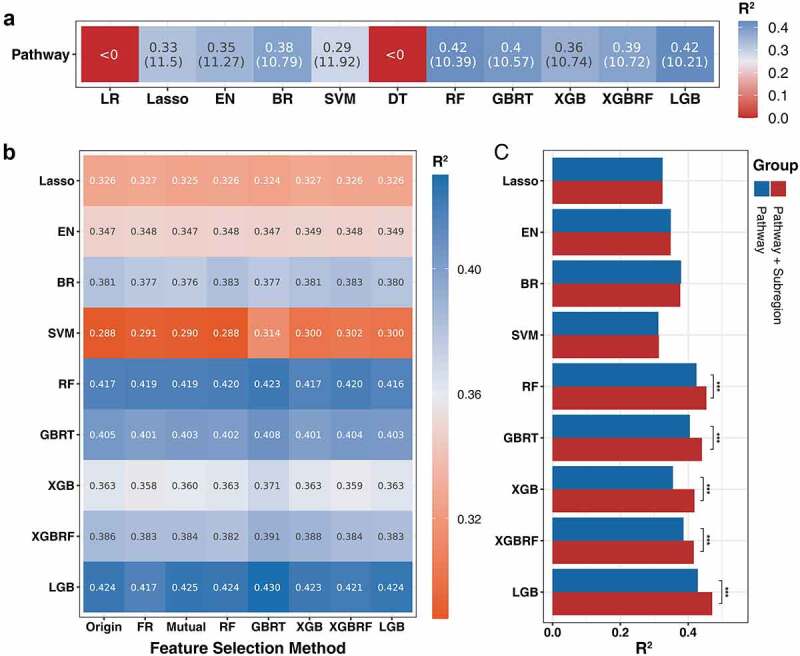
Performance of age prediction model based on pathways composition of gut microbiome. (a) The ability of different machine algorithms to predict the age of pathways composition (evaluation metric is R^2^ and MAE). (b) The impact of different feature selection algorithms on the performance of different models (filtered with age prediction ability, evaluation metric is R^2^). (c) The influence of extra subregion feature on the performance of age prediction (based on the pathway composition after feature selection).

As mentioned previously, we first judged whether this series of algorithms could predict age based on pathway data. After 10 times 5-fold cross-validation, the algorithms, with the exception of LR and DT, showed varying degrees of age regression capabilities (Figure 4a). Interestingly, compared to the taxonomic profiles, the pathway profiles had fewer feature dimensions but showed better prediction precision in most algorithms (the feature dimensions of the original species and pathway data were 904 and 468, respectively). However, although these two data types showed their best predictive capabilities in the LGB model, there was still a large performance gap between species and pathway. In general, in these distinct data types, tree-based algorithms demonstrated better predictive ability, while the LGB model had the best performance (R^2^ = 0.42, MAE = 10.21).

We next implemented the same feature selection strategy and the GBRT-based selection method has the least feature dimension without affecting the predictive performance of each model (Figure 4b, Supplementary Table S3). Thus, the GBRT-selected data were used for the subsequent model ensemble. We next compared the impact of an extra geographical feature on the model performance based on the screened data (Figure 4c). Similar to the phenomenon of species modeling, the tree-based methods were easier to learn the influence of subregion characteristics. In contrast, the geographic factors showed higher performance gains in the pathway modeling process.

### Ensemble and multi-view learning based on metagenomic data of gut microbiome

**Figure 5.**
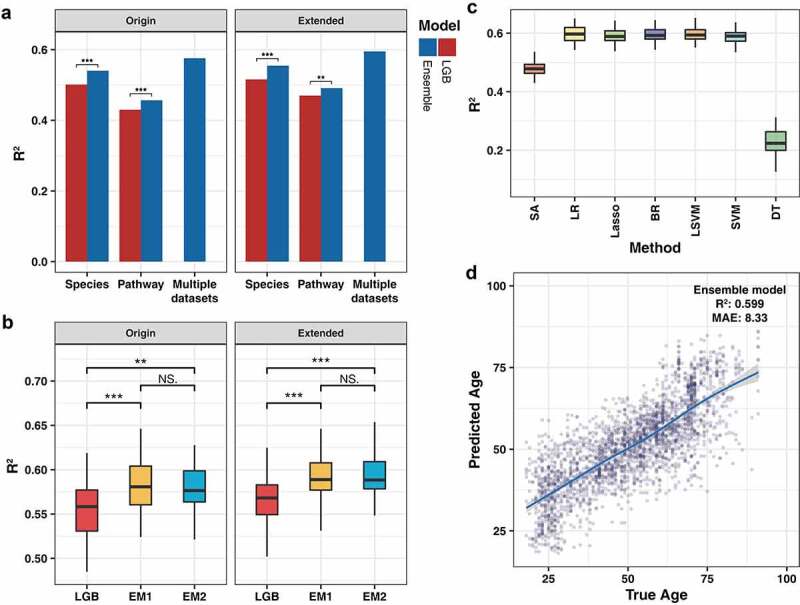
The predictive accuracy of modeling methods. (a) The accuracy of the different datasets analyzed by individual and ensemble model. (b) The impact of different data fusion methods on model prediction performance. (c) The predictive accuracy of different weighting methods on the extended dataset. There are significant differences in the prediction performance between the unlabeled groups. (d) Scatter plot of the true age and predicted age by the ensemble model (based on feature selected species and pathways data with extra subregion information). Origin, dataset after feature selection only; Extended, dataset after feature selection with extra subregion label. Paired Wilcoxon rank-sum test is used to analyze the difference between each group of data.

We next examined whether the fusion of multiple models can improve the prediction accuracy compared to a single model. To this end, nine pre-verified regression methods (Lasso, EN, BR, SVM, RF, GBRT, XGB, XGBRF and LGB) were applied to construct the ensemble model and LR was used as the weight learning algorithm of the generalizer. First, we compared the predictive accuracy of the single and ensemble algorithm with multiple data types (modeling using species set and pathway set respectively, and modeling using multiple datasets). To evaluate the performance, the LGB that showed the highest accuracy on both datasets was decided as the benchmark for the comparison. The result has shown that the ensemble method can significantly improve prediction accuracy in all data types, which emphasizes that the method was feasible (Figure 5a). Moreover, the result of multiple sets shows that the multi-view-based approach can further improve the performance.

It should be noted that, as the ensemble algorithm was simultaneous modeling of two datasets independently and then weighting results as the prediction outcome, and most of the current models were not designed to model multiple data sets simultaneously, meaning that the corresponding multiple datasets were not suitable for the LGB model. Therefore, due to the restriction of the data structure, it is hard to directly compare the performance difference between the ensemble model and LGB. To solve this problem, we adopted another data fusion way to compare the predictive ability between the LGB model and the ensemble model (Supplementary Figure S1). To realize the comprehensive utilization of the species and pathway data, we directly concatenated the two datasets. The metabolic pathway data of each sample were aggregated with the species data as additional features using the same method as the previous one that used the subregion feature. We used the concatenated dataset to construct the LGB and ensemble models (abbreviated as EM1) and compared them with the ensemble model that used the aforementioned integration strategy (EM2; train independently first, then weigh the results). As a result, we found that the expansion of features can also improve regression accuracy. Under this integration strategy, the performance of our ensemble model was still significantly higher than that of the LGB, and was not significantly different from that of the original ensemble method (Figure 5b).

We also tested dissimilar weights learning methods and judged the changes to the ensemble effect. The simple average (SA) result was used as the baseline for different weighting methods and the non-ensemble tree model was considered as the potential generalizer, including LR, Lasso, BR, SVM with linear kernel (LSVM), and SVM with non-linear kernel (SVM). The rationale behind this was to avoid overfitting of complex models in the generalization process and resulting in performance loss. We observed that the basic linear models (LR, Lasso, BR, LSVM and SVM) were able to obtain the ideal integration effect compared with the SA, and there was no significant difference between these methods (Figure 5c). It was noteworthy that the DT experienced severe performance degradation, with performance lower than the benchmark, which implies that the DT may have a serious overfitting problem. Combining the above results, we determined the simplest LR algorithm as the generalizer of the ensemble model, following which, basic learners can obtain an ideal performance. Our ensemble model demonstrated a stable predictive effect across all age ranges, with no abnormal prediction bias in any specific age range (Figure 5d).

### Interpreting age-related biomarkers of the gut microbiome based on the ensemble model

**Figure 6.**
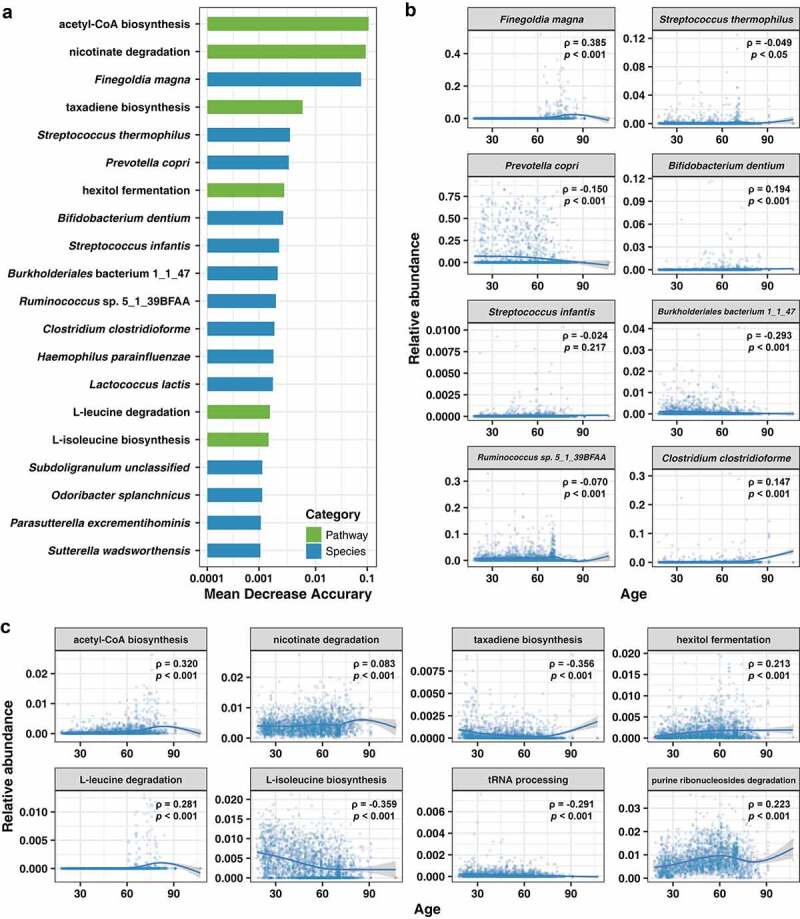
Aging-associated biomarkers have significant effect on model prediction performance. (a) Top 20 biomarkers with the highest effect on model prediction performance in ensemble model for prediction of age. (b) Top 8 most affected microbial species (c) Top 8 most affected microbial pathways. Correlation between species/pathways and age shown in terms of spearman’s rho (ρ). All p-values are adjusted for multiple comparisons using the Bonferroni correction and spline fit to the data is also shown (blue curve).

We next sought to interpret the ensemble model to identify the age-related features included species and pathway two aspects. The permutation feature importance (PFI) method was used to investigate the relationship between individual features and aging. We obtained a set of biomarkers that were significantly related to the aging process (Figure 6a). Among them, the species and pathways of the gut microbiome showed a different extent of influence on age prediction. We observed that a total of 102 microbial species and 41 metabolic pathways had a significant impact on age prediction (Supplementary Table S4). The most predictive factors were acetyl-CoA biosynthesis, nicotinate degradation and *Finegoldia magna*. The remaining factors included taxadiene biosynthesis, *Streptococcus thermophilus, Prevotella copri*, hexitol fermentation, *Bifidobacterium dentium* and *Streptococcus infants*.

Among these identified biomarkers, we found that many of the species and pathways showed specific trends with age. For example, *F. magna, B. dentium*, and *Clostridium clostridioforme* had increased abundance in the elderly, while *P. copri* and *Burkholderialse bacterium* 1_1_47 decreased in abundance with age (Figure 6b). Similar age distribution characteristics were observed in metabolic pathways; acetyl-CoA biosynthesis, nicotinate degradation and L-leucine degradation had a higher probability of enrichment in the gut with increasing age. In contrast, taxadiene biosynthesis, tRNA processing and L-isoleucine biosynthesis had a high possibility of function loss with increasing age (Figure 6c).

## Discussion

The development of machine learning algorithms has provided new opportunities for comprehensive and in-depth analysis of gut microbiome data, and has allowed us to associate the complex species and pathway composition of microorganisms with host states. In this study, we constructed and evaluated a novel ensemble modeling framework for universal age regression using a large-scale collection of metagenomic sequencing data of more than 2500 gut microbiomes. Our ensemble model achieved better prediction accuracy and higher data utilization capabilities than the currently used methods. It is worth noting that our study is unique in that we not only corrected the influence of location factors on the host flora, but we also integrated heterogeneity algorithms and multi-view learning in the field of gut microbiome research.

Precise construction of an aging clock based on the gut microbiome is important to be able to explain the role of gut flora in the aging process as it can guide subsequent anti-frailty interventions.^14,15^ The diversity of host background information has made the gut microbiome of each sample is unique, which will affect the accuracy of diagnosis based on the intestinal flora. Indeed, in some cases, this individual variation may even conceal the actual relationship between the microbiome and the host state.^16^ Therefore, invalid relevance caused by the distribution characteristics of background factors should be avoided to construct an accurate age prediction model. Previous studies have shown that correction of the confounding factors can improve the identification of the gut microbiome alterations and interpretation effects.^8^ Among the various factors, the geographical factor is considered to be the dominant confounding factor affecting the structure of the gut microbiome.^8,9^ Thus, we conducted a comprehensive meta-analysis that focused on whole age ranges (except minors) to extend the finding across a broader landscape of human aging; this may lead to the discovery of more general patterns of age-associated microbiome shifts.^17^ Simultaneously, we applied a two-step screening method to realize the decoupling of sample age and country. Our results showed that the above method can decouple geographical and age factors (Figure 2e). More importantly, the performance improvement achieved by adding the extra feature of subregion to the gut microbiome species or pathway composition was not caused by the direct relationship between the subregion and age, but by learning the unique flora characteristics of a different geographic area.

The whole genome of entire microbial communities can be profiled using modern high-throughput sequencing technology; however, the size and complexity of the corresponding flora sequence datasets also increase, and how to effectively manage, analyze, and integrate these high-dimensional big data has become a significant challenge. Recently, machine learning had been used to solve these problems because it is able to realize the interpretation of considering the interaction between flora to increase our understanding of the existing data structure.^18^ A plethora of microbiome studies has applied machine learning methods to established disease diagnosis models and explore the potential associations, such as for cancer, cardiovascular disease, and diabetes.^19–21^ Therefore, after deconvoluting the effect of regional factors, we made a systematic comparison between multiple heterogeneous model algorithms, in which each regressor was widely used in metagenomic data analysis.^22,23^ Each regressor may have a distinct output for the same input dataset due to different algorithm mechanisms. However, by taking advantage of the diversity of models, it is possible to construct a more accurate and less biased regression model. Thus, determining the base regressor that can accomplish age prediction was essential to achieve the model ensemble. In addition to considering the model algorithm, we further constructed the prediction model based on the metabolic function of the gut microbiome. It has been suggested that microbial function may be more informative and more conserved than taxonomic composition.^24^ The results of modeling on the two data types show that most models can realize age prediction based on gut microbiome metagenomics data, and the pathways demonstrated better results in most models (Figure 3a, Figure 4a). Additional tests were performed to compare the prediction accuracy of the corrections to the subregion factor, the results of which demonstrated that prediction precision can be further improved by considering host confounding factors. In summary, our integrative analysis reveals that both species and pathways are related to the human aging process and that adjusting for location factor can improve the identification of gut microbiome alterations in aging (Figure 3c, Figure 4c).

A stacking strategy was used to build the ensemble model in order to improve the accuracy and realize the comprehensive utilization of multiple sets of data. Ensemble methods have been widely used to enhance model prediction ability due to them being both effective and easy to implement. Although ensemble learning has been gradually applied to biological analysis such as cancer diagnosis based on gene expression,^25^ it is still limited to an individual model in the current microbiology researches. However, compared with a single algorithm in the modeling process, the corresponding cost of the ensemble algorithm leads to a substantial increase in the data calculation overhead. To reduce the running time of the model construction, different feature selection methods have been used to reduce feature dimensions. After feature filtering, the selected datasets show a reduced computational time and the model performance is slightly improved, so as to achieve a balance between performance and speed. The presented experiments strongly confirm the effectiveness of the integration method on three datasets (including species, pathways, and the combination of the two sets), and show that the multi-model ensemble has higher accuracy than any of the individual regressors acting solo on all of the datasets (Figure 5a). However, previous studies have tended to focus on species or genes to analyze the relationship between microorganisms and host states; thus, the metagenomic sequencing data, which contained a mass of information about the gut microbiome, were not able to be fully utilized. In this study, we did not limit to ensemble heterogeneous models, but also integrated different types of data simultaneously, which allowed us to achieve multi-view modeling. Our results demonstrate that the combination of species and pathways provides the model with better predictive ability, and confirm that comprehensive consideration of annotation data in different ways can describe the overall state of the host’s gut microbiome. We solved the limitations of the weak interpretation potential (low taxonomic annotation level for amplicon sequencing) and low forecast accuracy in previous studies.^6,7^ We also judged different data fusion strategies to prove that there is no performance distinction between the methods. The advantage of modeling each dataset before the ensemble is that decoupling datasets can improve data utilization compared to merging datasets first; indeed, independent modeling has better adaptability when the samples between multiple sets are not completely matched. However, the concatenated first strategy is unable to handle a large number of missing values, which is caused by the inconsistency of samples, such as the sample only provides the species composition and lacks functional annotations, or the sample has been sequenced whole-genome but the metabolome data is not measured. Therefore, these samples can only be discarding these samples directly and finally resulting in a waste of the data. The model first strategy can effectively solve this problem by utilizing all of the data to construct the models. In this strategy, only part of the matching data is needed for the final generalizer training to combine the separately trained models to maximize the use of datasets. Although this situation is not covered in this paper, high utilization of data is necessary, and although the omics technology for the intestinal flora is constantly developing and provides data support for related research, multi-omics data of the same sample, such as metagenomics, metatranscriptomics, and metabolomics, are still very scarce. In addition, most of the previous multi-omics studies have separately revealed patterns in each dataset; thus, it may be difficult to detect some fine-tuned structures that are not exposed by mining a single omics data type.^26^ Our calculation framework can realize the effective use of multi-omics data to analyze the comprehensive association between the gut microbiome and the host states, and may help to elucidate the complex mechanism of cross-omics.^27^

By relying on a more accurate age prediction model, we revealed potential biomarkers of aging by using comprehensive metagenomics annotation data with a wide age range. A total of 102 species and 41 pathways were considered to be closely related to the aging process. It is worth noting that many of the biomarkers are correlated with aging-related diseases or those more prevalent in the elderly (Figure 6a). For example, *Klebsiella pneumoniae* was one of the main causes of gram-negative bacterial infections of the bloodstream, and elderly patients have a higher risk of infection.^28^ Furthermore, *F. magna* and *P. copri* were correlated with arthritis, and consistent with this, the pathways related to acetate production (associated with arthritis) were also identified as biomarkers.^11,29,30^ In line with a previous study, a significant proportion of the species and pathways we identified were found to be related to the frailty of the elderly population, including *C. clostridioforme, Clostridium hathewayi, Clostridium bolteae, Clostridium leptum, Clostridiales* bacterium 1_7_47FAA and the pathway of pyruvate fermentation to acetone.^8^ In addition, some indicators may clarify the collective characteristics of the gut environment. Among the previously obtained biomarkers, we observed that the utilization of amino acids by the gut microbiome undergoes substantial changes with increasing age. This metabolic characteristic that leads to a reduction in amino acids may aggravate malnutrition in the aging process, which is not conducive to maintaining immune system function and prevent frailty.^31,32^ It should be noted that the consumption of branched-chain amino acids (BCAAs) is increased with age, especially the enrichment of the leucine metabolism and loss of the isoleucine synthesis pathway (Supplementary Table S4). BCAAs are believed to improve muscle protein synthesis, and related research has shown that the elderly population needs to consume higher levels of leucine;^33,34^ these findings are consistent with the key metabolic characteristics revealed by our analysis. In addition, pathways related to tryptophan biosynthesis are also believed to be related to aging. The kynurenine pathway is considered to be the main tryptophan metabolism pathway in humans. The kynurenine is neurotoxic and can directly compromise mitochondria, which in turn leads to aging-related inflammation.^35^ The synthesis of tryptophan in the intestinal flora may lead to the accumulation of kynurenine in the body. These age-related change eventually may lead to disease. These age-associated biomarkers clarify the potential role of the gut microbiome in the aging process, and the integration method allowed us to rank different types of data to achieve more refined directional control for aging.

The current study consists of a multi-view data-adapted ensemble machine learning framework. Through the integration of heterogeneous models and data, we observed broad patterns of gut microbiome changes in aging and clarified the impact of the species and functions in this process. Future studies should focus on further improving the collection of big data on the gut microbiome. Although we have removed some host confounding factors in this study, more high-quality and diverse data are necessary to explore the in-depth associations between gut bacteria and age. The machine learning framework should also be improved. Indeed, new algorithms are constantly being proposed, especially neural network models, which have shown considerable predictive potential in other fields.^36^ Through improvements in methods, it will be possible to extend data to ensure the reliability of the conclusion and maximize its utility.

## Materials and methods

### Sample cohorts

The data used in this analysis comes from a pre-processed public metagenome data from curatedMetagenomicData.^37^ We first selected stool samples and subsequently removed the samples that contained incorrectly annotated information. From these datasets, we deleted samples having age less than 18 years of age, a total of 4478 stool samples from 31 cohorts are included in this study (Supplemental Table S1).

### Effect of host-associated factors

In order to explore the impact of metadata between different datasets, we considered the reliable and non-redundant metadata which is defined as missing values is less than 40% and not associated with existing metadata (E.g. Age and Age-category), and finally 7 metadata remained, divided into 5 covariates (non-westernized, age, gender, country, and BMI) and 2 confounding factors (sequencing platform, DNA extraction kit). And for subsequent data analysis, we regrouped country into subregion level (based on United Nations subregions, standard country or area codes for statistical use, M49).

Permutational multivariate analysis of variance using distance matrices (PERMANOVA, provided by R package vegan::adonis) was used to quantitatively evaluate the impact of metadata. The PERMANOVA analysis for each covariate was using the following formula: adonis(dissimilarities ~ covariate + sequencing platform + DNA extraction kit, permutations = 9999). Bray-Curtis dissimilarity was used in PERMANOVA to judge the beta diversity of the species and pathway samples, and the diversity distances are calculated by R packages (vegan::vegdist). In addition, Bonferroni was applied for p-value correction.

To filter a subset which was no correlation between geographical and age, we first constructed a random forest age prediction model based on full subregion labels (encode by sklearn OneHotEncoder). Next, we removed the corresponding subregion data in descending order of feature importance score until one area remained. In each epoch, a random forest model was constructed with screened data, and 10 times 5-fold cross-validation was used to judge the association.

### Machine learning regressor construction

To achieve an effective model ensemble, heterogeneous machine regression models were constructed, including Linear Regression (LR), Lasso, Elastic Net (EN), Bayesian Ridge (BR), Support Vector Machine (SVM), Decision Tree (DT), Random Forest (RF), Gradient Boosted Regression Trees (GBRT), eXtreme Gradient Boosting (XGB) and LightGBM (LGB). Before construct machine learning models, the zeros in species and pathway profiles were replaced with a very small number to suit logarithmic conversions (using the multiplicative_replacement function implemented in scikit-bio, http://scikit-bio.org/). Next, use the centered log-ratio (CLR, using the clr function implemented in scikit-bio) to transform the preprocessed profiles.

For each algorithm, built regression model based on species and metabolic pathway data. 10 times 5-fold cross-validation was performed to avoid random errors, R^2^ and mean absolute error (MAE) were used as the performance evaluation metrics. Among them, LR, Lasso, EN, BR, SVM, DT, RF and GBRT models are constructed using packages of the scikit-learn,^38^ XGB and XGBRF are constructed using xgboost package^39^ and LGB model is constructed using lightgbm package.^40^

### Feature selection method

Feature selection was performed to improve prediction accuracy and decrease feature dimension which helped to reduce the computational cost, contain Univariate linear regression tests (f_regression, FR), Mutual information estimation and model-based selection method (RF, GBRT, XGB, XGBRF and LGB). For each feature selection method, the filtered data was applied to each regressor with 10 times 5-fold cross-validation to avoid random errors, and the average R^2^ score was used as an evaluation indicator. We next used paired Wilcoxon rank-sum test to compare the performance changes between the original features and the filtered features, and the *p*-value was corrected by Bonferroni.

The feature selection method was evaluated by the number of models that performance was significantly different after selection, the method with the least number of models with the lowest performance degradation is the optimal algorithm. In the case of multiple methods with the same counts, the number of models with the performance increase and the feature dimension as the secondary screening basis.

### Multiple regressor ensemble and multi-view learning

A stacking ensemble strategy was utilized to construct a powerful meta-learner and the stacking process includes two stages. In the first stage (Figure 1a), the training set is randomly split into k-fold (5-fold was used in this study). Used four of the subsets to construct machine learning model, and the rest as the validation set, repeat the above process until all the subsets are traversed. Finally, we obtain a predicted value dataset corresponding to the entire training set named “Predicted Training Set”. Simultaneously, we get five prediction outcomes by test sets, calculate the mean of the result and named “Predicted Test Set”.

In the second stage (Figure 1b), The prediction results of heterogeneous base learners in stage one are fed to stage two as the input, a generalizer was trained to combine the predictions from the first stage and optimized to form a final prediction output. In this training process, LR was used as the generalizer to learn the weights of different base learns. The weighted result of the test set was used as the result of the final integrated regression prediction model.

For multi-view learning, the integration of different data types was performed in the second stage. After performed the training of each model in the first stage on different types of datasets and obtained their predicted training set. Next concatenated each set to obtain the merged set for weights learning and perform the same process as mentioned above.

### Feature interpretation for ensemble model

After constructed the ensemble model, in order to determine the importance of each feature in the prediction process, the permutation feature importance (PFI) was used to measure individual feature importance which evaluates by eliminating a single feature (randomly shuffle the feature 50 times) to calculate the extent of change in the prediction effect (R^2^ score reduction as the metric and the feature which has a positive effect of R^2^ score was not considered). Based on multiple repeated calculation results, paired Wilcoxon rank-sum test was used to compare the prediction performance with the original model which without feature replacement. Features that are significantly different from baseline data are considered as potential biomarkers and ranked by the extent of influence on the prediction performance. Furthermore, Spearman’s rho test was applied to judge the association between the biomarkers and age. All *p*-values were Bonferroni-corrected.

## Supplementary Material

Supplemental Material

## Data Availability

The data presented in this study are are available in GitHub: https://github.com/hcwang-jn/gut-ensemble
