# Supplementary material for: Human gut microbiome aging clocks based on taxonomic and functional signatures through multi-view learning
Source: Gut Microbes. 2022 Jan 18;14(1):2025016. doi: 10.1080/19490976.2021.2025016 (PMC8773134; doi:10.1080/19490976.2021.2025016)
Supplement: Supplemental Material [file KGMI_A_2025016_SM4749.zip › supplementary/Supplementary_figure_1.docx]

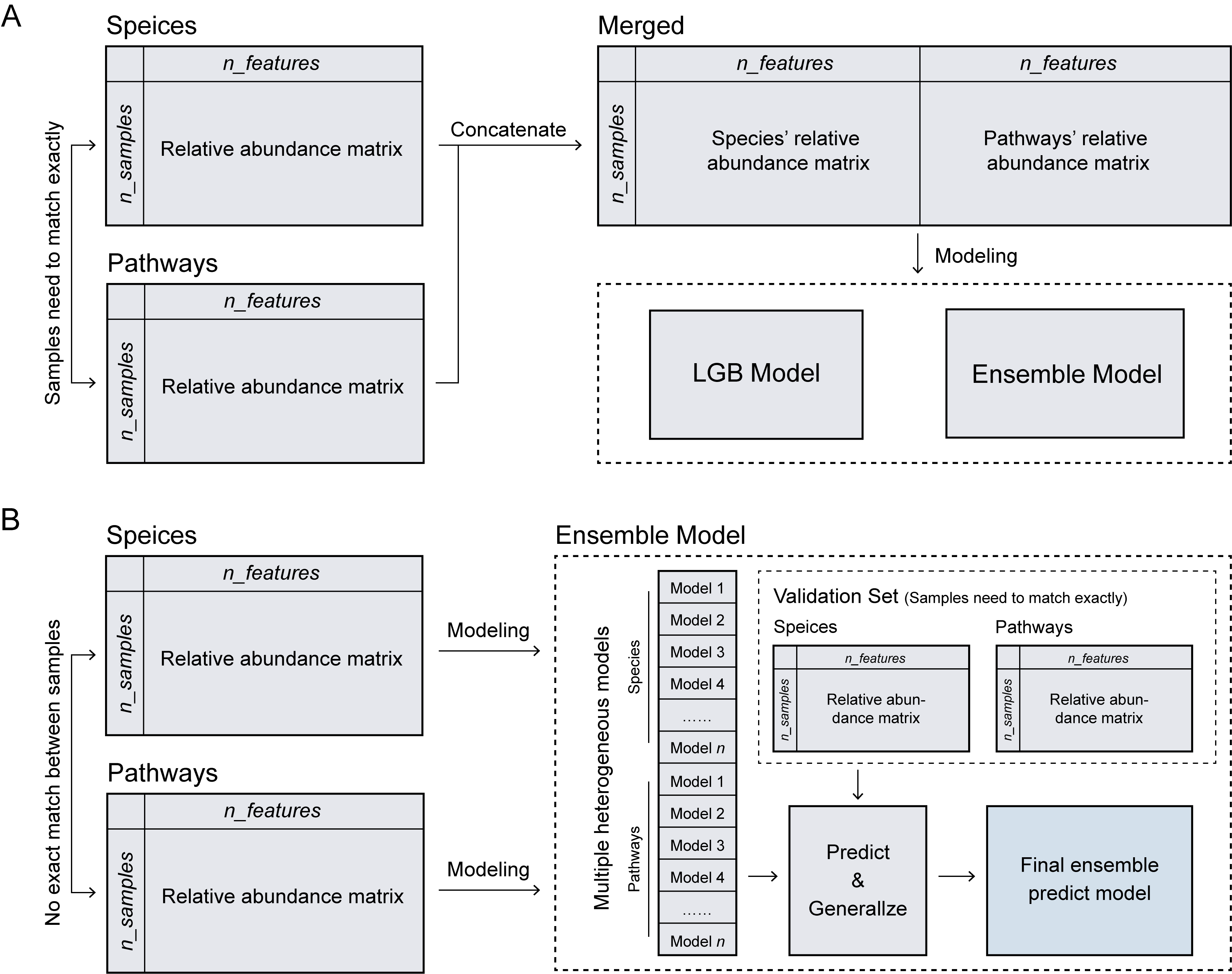


Supplementary Fig S1. The strategies of data integration. (A) Directly concatenate of different data sets. (B) First construct model on each data set and then integrate the model prediction results (The default ensemble model construction strategy). In these two data fusion processes, the method of establishing the integrated model is essentially the same (the mechanism details are described in the Method), here we mainly emphasized the differences in data set requirements in different mechanisms.
